# Differential Modulation of Extracellular Matrix- and Longevity-Related Gene Expression by Calcium Hydroxylapatite Formulations

**DOI:** 10.3390/life16091400

**Published:** 2026-08-25

**Authors:** Alessandra Haddad, Maria Cláudia Almeida Issa, Luiz Tonon, Beatriz Domenici de Oliveira, Bibiana Franzen Matte, Glaucia Maria Machado Santelli, Renata Viana

**Affiliations:** 1 Department of Plastic Surgery, Federal University of São Paulo, São Paulo 04532-012, SP, Brazil; 2 Department of Clinical Medicine, Fluminense Federal University, Niterói 24033-900, RJ, Brazil; 3 Private Practice, São Paulo 04089-001, SP, Brazil; 4 Ilikia Brasil, São Paulo 01427-000, SP, Brazil; 5 Nucleo Vitro, Porto Alegre 91040-600, RS, Brazil; 6 Department of Pharmacology, Institute of Biomedical Sciences, University of São Paulo (USP), São Paulo 05508-900, SP, Brazil

**Keywords:** calcium hydroxylapatite, dermal fibroblasts, gene expression, biostimulation

## Abstract

Background/Objectives: Calcium hydroxylapatite (CaHA) is widely used in aesthetic medicine as a biostimulatory dermal filler. While its ability to induce neocollagenesis is well established, its broader transcript-level effects on fibroblast biology under aging-like conditions remain incompletely characterized. This study aimed to compare the gene-expression responses induced by three commercially available CaHA formulations in an EX-527-induced aging-like human dermal fibroblast model. Methods: An aging-like fibroblast phenotype was induced in primary human dermal fibroblasts using the SIRT1 inhibitor EX-527, which was maintained in culture throughout the CaHA treatment period. Cells were exposed to three commercially available CaHA formulations (D, R, S) for 72 h. Gene expression was assessed by RT-qPCR, evaluating markers related to extracellular matrix remodeling, *Ki67* expression, inflammatory and regulatory cytokine signaling, mitochondrial stress response and energy metabolism, and longevity-associated genes. Results: All CaHA formulations induced upregulation of *COL1A1* and *Ki67*, with greater responses observed in samples S and R. *ELN* expression was more selectively increased, particularly in sample S. Distinct cytokine gene-expression profiles were observed, with sample D associated with increased *TNF-α* expression, while samples R and S demonstrated reduced *TNF-α* and increased *IL-10* expression. Genes associated with mitochondrial stress response and energy metabolism, including *PGC-1α*, *SOD2*, and *PINK1*, were upregulated across groups, with the highest induction generally observed in sample S. *FOXO3*, *SIRT1*, and *SIRT3* expression also increased following treatment, with sample S showing the most pronounced response for *FOXO3* and *SIRT3*. Conclusions: These transcript-level findings suggest that CaHA exposure is associated with differential gene-expression responses involving markers related to extracellular matrix remodeling, inflammatory and regulatory signaling, mitochondrial stress response, and longevity-associated pathways. Functional and protein-level validation will be required to determine whether these transcriptional changes translate into meaningful biological effects in more complex models of aging skin.

## 1. Introduction

Skin aging is a multifactorial biological process, characterized by progressive structural and functional deterioration of the dermal extracellular matrix (ECM) [1,2,3]. A key feature of dermal aging is the progressive alteration of the collagen-rich extracellular matrix, including reduced fibrillar collagen content, impaired fibroblast–collagen interactions, and collagen fibril fragmentation, which is particularly prominent in photoaged skin [1,3,4,5]. Consequently, these structural changes are closely associated with functional alterations in dermal fibroblasts, such as reduced proliferative capacity and decreased synthesis of extracellular matrix components [2,3,6,7].

Fibroblast function relies on interaction with the extracellular matrix [4,5]. Mechanical anchorage to collagen fibrils maintains cytoskeletal tension and supports signaling pathways that regulate collagen production and cellular homeostasis. When this interaction is disrupted, collagen synthesis is reduced, and fibroblast function is impaired [4,5]. Loss of fibroblast–matrix interaction contributes to reduced collagen synthesis and extracellular matrix remodeling, while cellular senescence, mitochondrial dysfunction, oxidative stress, and low-grade inflammation are closely linked to dermal aging [3,4,7,8].

Recent reviews further emphasize dermal fibroblast senescence as a central component of skin aging, integrating extracellular matrix dysfunction, SASP activity, mitochondrial impairment, oxidative stress, and altered intercellular signaling [9]. Cellular senescence has also been increasingly discussed as a molecular target in skin aging and skin disease, reinforcing the relevance of senescence-associated in vitro models while also highlighting their translational limitations [10]. Furthermore, mitochondrial dysfunction and increased oxidative stress exacerbate these changes by impairing energy metabolism and promoting chronic low-grade inflammation [6,7,8].

At the molecular level, pathways associated with cellular longevity play a key role in regulating fibroblast function and tissue homeostasis. Among these, the sirtuin family of NAD^+^-dependent deacetylases, particularly *SIRT1*, has been extensively implicated in the regulation of stress responses, mitochondrial function, and cellular survival [11,12].

Within this context, aesthetic medicine has shifted toward the use of biomaterials that interact with local tissue and modulate biological responses, including extracellular matrix remodeling, rather than relying solely on volumetric correction. Among these, calcium hydroxylapatite (CaHA)—a synthetic material of calcium phosphate microspheres suspended in a carrier gel—is widely used for soft tissue augmentation and biostimulation due to its favorable biocompatibility [13,14,15,16,17].

Experimental evidence shows that CaHA can directly affect fibroblast behavior by stimulating collagen production and extracellular matrix remodeling through cell–material interactions. Recent hydroxyapatite microsphere/hydrogel data also support the relevance of HAp-based particles in skin-regeneration models, with reported effects on fibroblast functionality, calcium signaling, motor-protein pathways, and ECM remodeling [18]. These effects appear to be partly driven by direct contact between CaHA microspheres and dermal fibroblasts, which may activate pathways involved in tissue repair and regeneration [17,19]. Recent in vitro studies also suggest that CaHA-induced fibroblast responses may extend beyond fibrillar protein synthesis and may include modulation of longevity-associated gene expression [20].

Despite its broad clinical use, commercially available CaHA-based products may differ in physicochemical, manufacturing-related, and handling characteristics that could influence cellular responses. Beyond physicochemical properties, differences in microsphere characteristics and other properties may affect cellular responses, such as inflammatory responses [21]. However, controlled experimental data comparing these effects in similar commercially available biomaterials remains limited.

Therefore, the present study aimed to address gaps in comparative data by evaluating the in vitro performance of different commercially available CaHA biomaterials. Using an EX-527-induced aging-like fibroblast model, we investigated CaHA-induced transcriptional responses under conditions that reproduce selected senescence-associated features of dermal aging. By analyzing gene expression related to extracellular matrix remodeling, cellular proliferation, inflammatory response, and pathways associated with cellular homeostasis, we intend to clarify whether CaHA formulations differentially influence key mechanisms of dermal aging and tissue remodeling.

Through this approach, we sought to characterize exploratory transcript-level differences among formulations and to generate hypotheses for future mechanistic validation.

## 2. Materials and Methods

### 2.1. Calcium Hydroxylapatite Samples

Three CaHA-based dermal fillers were evaluated: Diamond Intense Rennova (Lot F-2249112; Dr. Korman Laboratories Ltd., Kiryat Bialik, Israel), Radiesse (Lot A00162770; Merz North America, Franksville, WI, USA), and Stiim (Lot S2Q25025; CGBio Co., Seoul, Republic of Korea). Samples were labeled D (Diamond Rennova), R (Radiesse), and S (Stiim). All were handled according to the manufacturer’s instructions.

To enable in vitro application, all materials were diluted in culture medium to obtain a stock concentration of 10 mg/mL, followed by further dilution to a non-cytotoxic working concentration of 0.1 mg/mL, previously determined as non-cytotoxic based on pilot standardization assays using the MTT colorimetric method [22,23].

### 2.2. Cell Culture and Induction of Senescence-Associated Phenotype

Primary human dermal fibroblasts were obtained from a healthy donor under an Ethics Committee/IRB-approved protocol (CAAE #59124916.6.0000.5327) and were used for all experiments. Cells were cultured in Dulbecco’s Modified Eagle’s Medium (DMEM; Gibco, Thermo Fisher Scientific, Inc., Waltham, MA, USA) supplemented with 10% fetal bovine serum (FBS; Gibco, Thermo Fisher Scientific, Inc., Waltham, MA, USA) and 1% penicillin/streptomycin solution (Penicillin-Streptomycin; Gibco, Thermo Fisher Scientific, Inc., Waltham, MA, USA) under standard controlled conditions at 37 °C and 5% CO_2_.

To model selected features of an aging-like dermal fibroblast state, a senescence-associated phenotype was pharmacologically induced in primary human dermal fibroblasts (passage 23) by exposure to EX-527, a selective SIRT1 inhibitor widely used as a pharmacological tool to inhibit *SIRT1* catalytic activity [24,25,26], at a concentration of 10 μM for 48 h [24,25,26]. The induction of this phenotype was supported at the transcript level by RT-qPCR analysis of *FOXO3*, *SIRT1*, and *SIRT3*, comparing untreated control fibroblasts with EX-527–exposed fibroblasts.

To provide additional phenotypic evidence of the aging-like cellular state, senescence-associated β-galactosidase (SA-β-gal) staining, a widely used biomarker of cellular senescence, was also performed according to the laboratory’s standard protocol [27]. Representative photomicrographs were obtained by light microscopy to compare untreated control fibroblasts and EX-527–treated fibroblasts. All procedures involving human-derived cells were conducted in accordance with institutional ethical standards.

### 2.3. Treatment Protocol

After induction of the aging-like phenotype, EX-527 was maintained in the culture medium throughout the 72-h treatment period. Fibroblasts were seeded in 24-well culture plates at a density of 3 × 10^4^ cells/well and treated with CaHA formulations (D, R, S) at 0.1 mg/mL in the continued presence of EX-527. The control group consisted of EX-527-exposed fibroblasts maintained under identical culture conditions, including continued exposure to EX-527, but without treatment with CaHA formulations. Cultures were incubated for 72 h at 37 °C and 5% CO_2_ prior to molecular analysis.

The single non-cytotoxic working concentration and 72-h exposure period were selected as standardized screening conditions to enable direct comparison among CaHA formulations under identical experimental parameters, rather than to define an optimal, physiological, or clinically equivalent exposure condition.

### 2.4. Gene Expression Analysis (RT-qPCR)

Following the 72-h incubation, total RNA was extracted using the TRIzol^TM^ reagent (Invitrogen, Thermo Fisher Scientific, Carlsbad, CA, USA), with quantity and purity being evaluated. Complementary DNA (cDNA) was synthesized from the extracted RNA via a reverse transcriptase reaction. Quantitative real-time PCR (RT-qPCR) was then performed using SYBR Green chemistry to analyze the expression of genetic markers categorized according to biological function (Table 1).

Gene expression levels were normalized to β-actin (*ACTB*) as the endogenous control, and relative expression was calculated using the 2^−ΔΔCt^ method. *ACTB* stability was assessed across experimental conditions, and no significant variation in raw Ct values was observed among groups. Results were expressed as fold-change relative to the untreated EX-527-exposed control group, which was normalized to 1.0. Primer sequences used for RT-qPCR analysis are provided in Supplementary Table S1.

### 2.5. Statistical Analysis

Data processing was performed using Microsoft Excel and GraphPad Prism version 9 (GraphPad Software, San Diego, CA, USA). All analyses were performed from three independent biological experiments. Results are presented as mean ± standard deviation (SD). Data normality was assessed prior to statistical testing. For normally distributed data, comparisons among groups were performed using one-way analysis of variance (ANOVA), followed by Bonferroni’s post-hoc test for multiple comparisons. A *p*-value below 0.05 was considered statistically significant. No formal sample size calculation was performed, as this was an exploratory in vitro study designed to compare transcriptional responses among CaHA formulations under standardized experimental conditions.

## 3. Results

### 3.1. Characterization of the Senescence-Associated/Aging-like Fibroblast Phenotype

To establish a senescence-associated/aging-like fibroblast phenotype, human dermal fibroblasts were exposed to the SIRT1 inhibitor EX-527 (Selisistat). The induction of a senescence-associated phenotype was evaluated by assessing the expression of key longevity-associated genes (*FOXO3*, *SIRT1*, and *SIRT3*), using non-treated cells as the reference control, and by representative senescence-associated β-galactosidase (SA-β-gal) staining.

**Figure 1:**
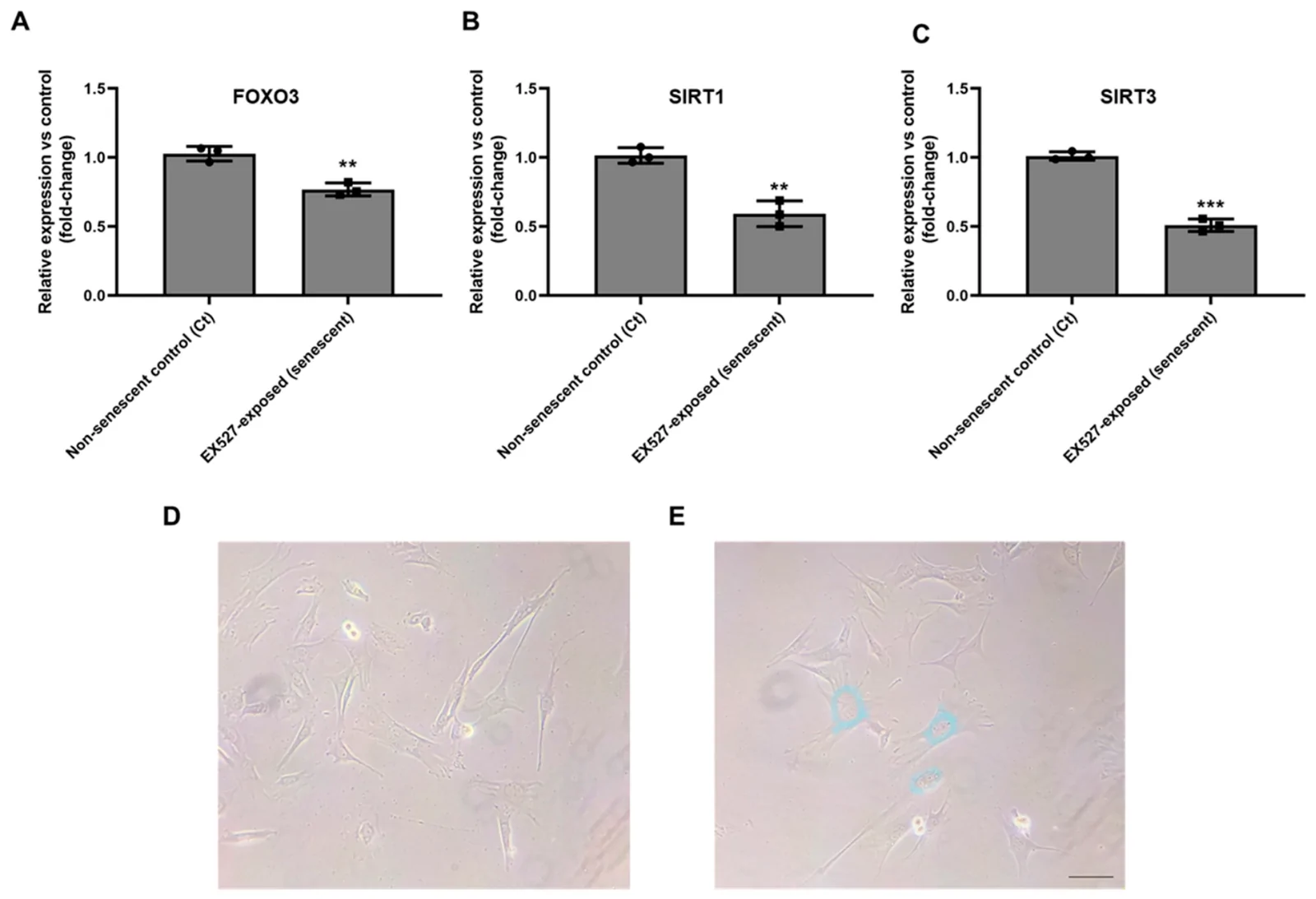
Characterization of the EX-527-induced aging-like fibroblast phenotype. Relative expression of (**A**) *FOXO3*, (**B**) *SIRT1*, and (**C**) *SIRT3* in EX-527-exposed fibroblasts compared with non-exposed controls (control normalized to 1.0). Data are shown as mean ± SD. ** *p* < 0.01, *** *p* < 0.001 versus control. (**D**) Representative senescence-associated β-galactosidase (SA-β-gal) staining of control fibroblasts and (**E**) EX-527-treated fibroblasts, showing increased β-gal-positive staining after EX-527 exposure. The scale bar corresponds to 50 µm.

Compared to non-senescent controls, EX-527-treated fibroblasts exhibited a consistent reduction in gene expression levels across all evaluated markers. *FOXO3* expression decreased by 23.3 ± 6.5%, *SIRT1* by 43.3 ± 8.2%, and *SIRT3* by 50.1 ± 6.2%. All reductions were statistically significant (*FOXO3* and *SIRT1*: ** *p* < 0.01; *SIRT3*: *** *p* < 0.001), supporting the establishment of a senescence-associated/aging-like molecular profile. Representative SA-β-gal staining further showed increased β-gal-positive staining in EX-527-treated fibroblasts compared with non-senescent controls, providing additional qualitative phenotypic support for the senescence-associated/aging-like cellular state (Figure 1D).

### 3.2. Effects of CaHA on Structural Dermal Matrix Gene Expression

*COL1A1* and *ELN* expression were quantified to assess treatment-induced changes in structural dermal matrix gene expression in aging-like dermal fibroblasts after 72 h of exposure. Gene expression levels were normalized to the untreated senescent control group.

**Figure 2:**
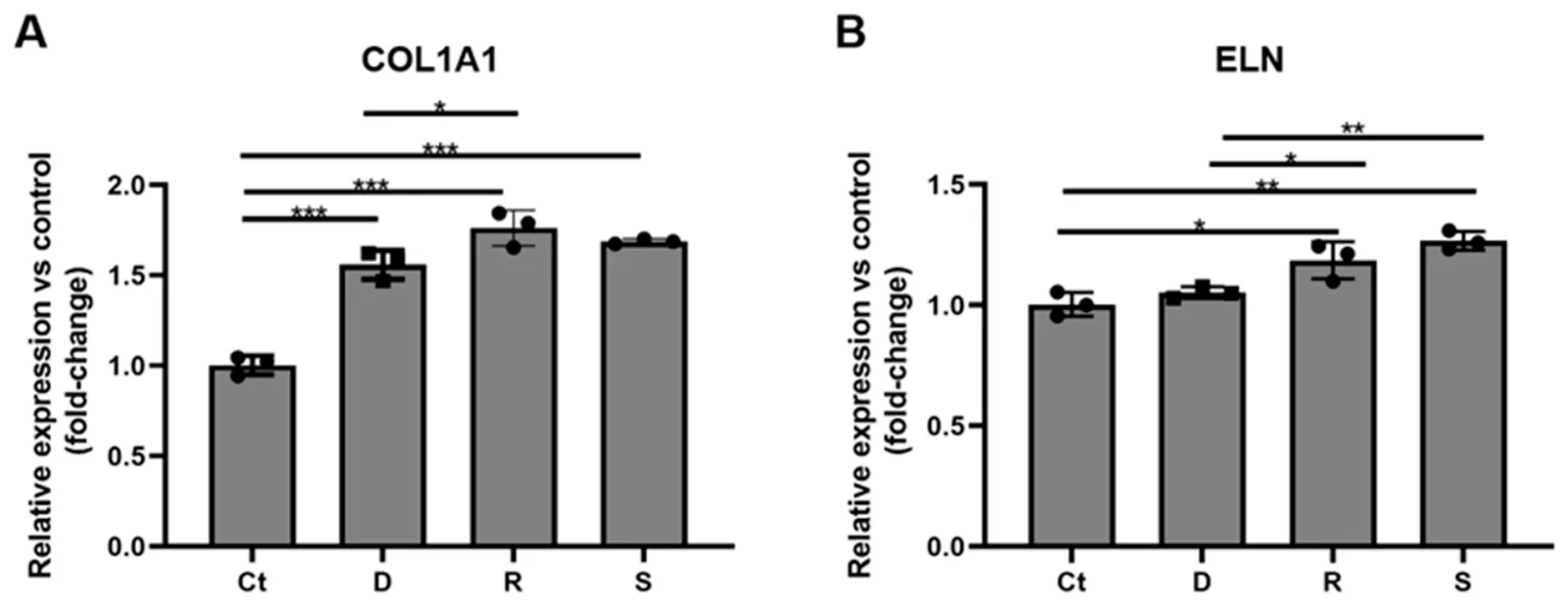
Relative gene expression of structural matrix markers (**A**) collagen type I and (**B**) elastin across experimental groups. Data are shown as fold-change relative to the untreated senescent control (normalized to 1.0). Horizontal bars indicate statistically significant differences between groups (* *p* < 0.05; ** *p* < 0.01; *** *p* < 0.001).

For the evaluation of *COL1A1*, all CaHA formulations induced a marked increase in gene expression relative to control. Sample R showed an increase of 74.4 ± 5.6%, followed by sample S (+68.8 ± 1.4%) and sample D (+54.4 ± 8.8%). Statistical analysis confirmed significant differences between the control and all treated groups, with no significant difference between samples R and S, although a significant difference was observed between R and D samples (Figure 2A).

Elastin expression exhibited a distinct response pattern across formulations. Sample S showed the highest increase (+26.7 ± 4.3%) followed by Sample R (+18.5 ± 7.8%), whereas sample D showed a comparatively modest effect (+3.81 ± 2.4%). Samples R and S showed a significant increase in *ELN* expression compared with the control group, whereas sample D showed a modest, non-significant increase. Sample D differed significantly from both R and S (Figure 2B).

The differential magnitude of collagen and elastin upregulation across formulations suggests variability in the ability of CaHA materials to stimulate extracellular matrix-related gene expression in aging-like fibroblasts, with a consistent pattern of greater transcriptional response observed in samples S and R compared to D.

### 3.3. Effects of CaHA on Ki67 Gene Expression

**Figure 3:**
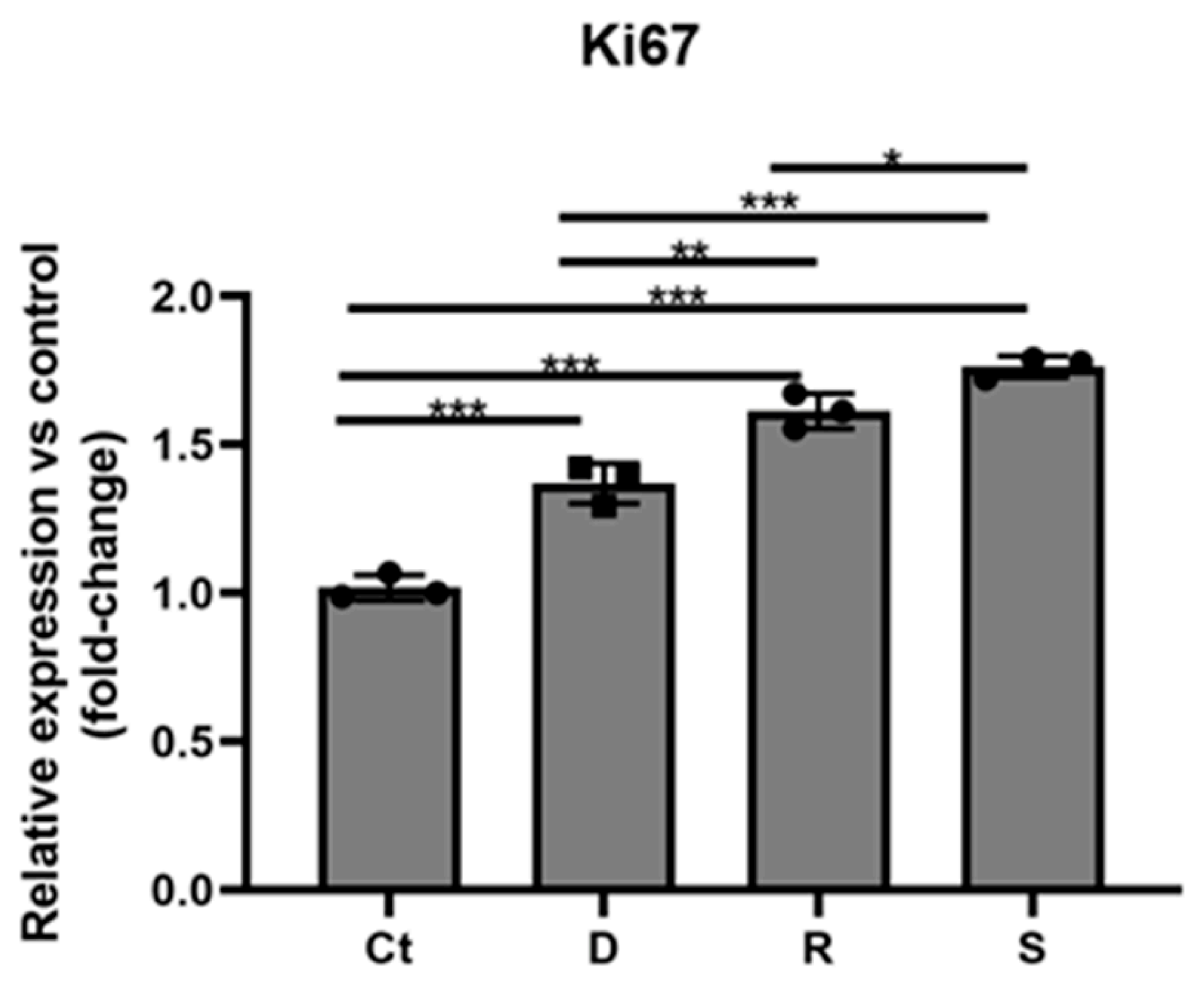
Relative gene expression of *Ki67* across experimental groups. Data are shown as fold-change relative to the untreated senescent control (normalized to 1.0). Horizontal bars indicate statistically significant differences between groups (* *p* < 0.05; ** *p* < 0.01; *** *p* < 0.001).

*Ki67* expression was quantified to assess treatment-related changes in a cell-cycle-associated transcriptional marker. All CaHA formulations induced a significant increase in *Ki67* expression compared to the untreated senescent control. Upregulation was highest in S (+74.7 ± 2.6%), followed by sample R (+58.5 ± 7.0%) and D (+34.6 ± 5.9%). Statistical analysis confirmed significant differences between the control and all treated groups, as well as significant differences among the treated groups (Figure 3).

The graded response across formulations indicates differential *Ki67* transcriptional responses to the tested CaHA materials under aging-like conditions, with samples S and R showing a more pronounced response than sample D.

### 3.4. Inflammatory and Regulatory Response Genes

Inflammatory cytokine gene expression was quantified to evaluate treatment-related modulation of pro-inflammatory (*IL-6*, *TNF-α*) and regulatory (*IL-10*, TGF-β) markers.

**Figure 4:**
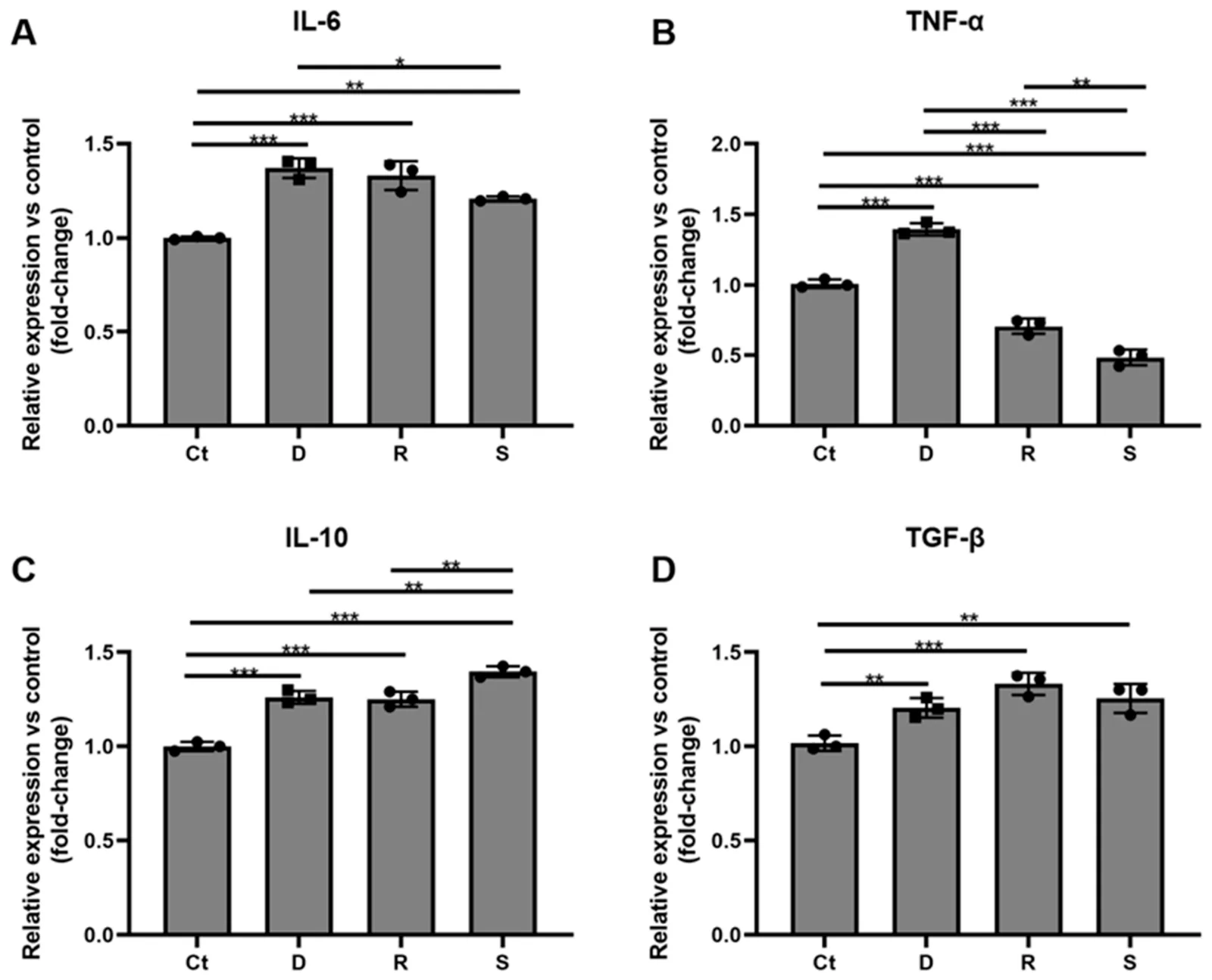
Relative gene expression of inflammatory cytokines across groups. (**A**) *IL-6*, (**B**) *TNF-α*, (**C**) *IL-10* and (**D**) TGF-β. Data are shown as fold-change relative to the untreated control (normalized to 1.0). Horizontal bars indicate statistically significant differences between groups (* *p* < 0.05; ** *p* < 0.01; *** *p* < 0.001).

Pro-inflammatory *IL-6* expression increased across all groups, with sample D showing the highest upregulation (+33.7 ± 5.9%), followed by sample R (+30.5 ± 6.3%) and sample S (+20.8 ± 1.1%). All treatments differed significantly from the control group, with a significant difference observed between samples D and S (Figure 4A).

*TNF-α* expression exhibited a distinct pattern across formulations. Sample D demonstrated an increase (+38.3 ± 4.7%), whereas samples R (−30.4 ± 5.2%) and S (−52.3 ± 3.3%) showed a reduction in pro-inflammatory *TNF-α* expression relative to control. All treatments differed significantly from the control group (Figure 4B).

Anti-inflammatory *IL-10* expression was upregulated in all groups, with sample S showing the highest increase (+39.7 ± 2.9%), compared to sample D (+25.2 ± 3.3%) and sample R (+24.1 ± 3.7%). All treatments differed significantly from control, and expression in sample S was higher than in both D and R (Figure 4C).

*TGFB1* expression also increased across all groups, with sample R demonstrating the highest upregulation (+31.8 ± 5.5%), followed by sample S (+24.3 ± 6.3%) and sample D (+20.3 ± 4.7%). All treatments differed significantly from the control group (Figure 4D).

Overall, CaHA formulations differentially modulated inflammatory and regulatory cytokine expression, with distinct response profiles observed across materials. While sample D showed increased expression of pro-inflammatory markers, samples S and R demonstrated a cytokine profile characterized by lower *TNF-α* expression and increased *IL-10* expression, suggesting a more regulated inflammatory gene-expression profile.

### 3.5. Effects of CaHA on Longevity-Associated Gene Expression

The expression of *FOXO3*, *SIRT1*, and *SIRT3*, genes associated with cellular longevity and stress response, was assessed in senescence-associated fibroblasts after exposure to CaHA formulations.

**Figure 5:**
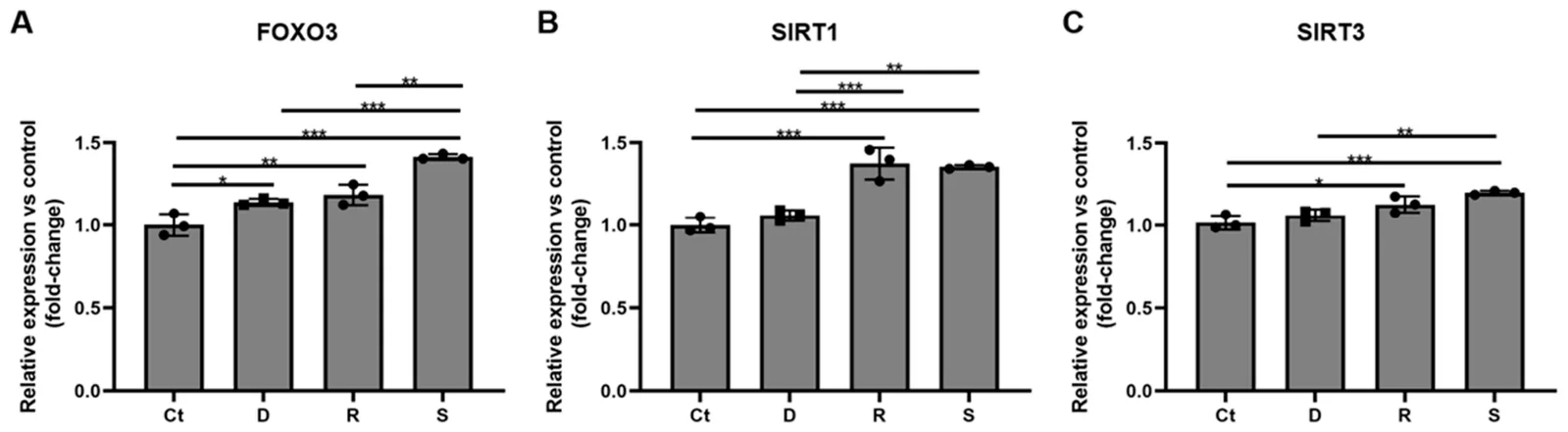
Relative gene expression of longevity-associated markers across groups. (**A**) *FOXO3*, (**B**) *SIRT1*, and (**C**) *SIRT3*. Data are shown as fold-change relative to the untreated control (normalized to 1.0). Horizontal bars indicate statistically significant differences between groups (* *p* < 0.05; ** *p* < 0.01; *** *p* < 0.001).

*FOXO3* expression increased in all groups, with sample S demonstrating the highest upregulation (+41.4 ± 1.7%), compared to sample R (+18.4 ± 6.1%) and sample D (+14.0 ± 2.1%). All treatments differed significantly from the control group, and expression in sample S was significantly higher than in both R and D (Figure 5A).

*SIRT1* expression showed a modest, non-significant increase in sample D (+5.9 ± 3.2%), while significantly higher increases were observed in samples R (+37.4 ± 9.6%) and S (+35.4 ± 1.1%) compared with the control group. Sample D differed significantly from both R and S, whereas no clear difference was observed between R and S (Figure 5B).

*SIRT3* expression increased across treatment groups, with sample S showing the highest upregulation (+20.0 ± 1.1%), followed by sample R (+12.3 ± 4.5%) and sample D (+5.7 ± 3.5%). Samples R and S showed significant increases compared with the control group, whereas sample D showed a modest, non-significant increase (Figure 5C). Overall, CaHA formulations modulated the expression of genes associated with cellular stress response and longevity-related pathways, with a consistent pattern of greater biological activity observed in samples S and R compared to sample D, and a more pronounced effect of sample S in *FOXO3* and *SIRT3* expression.

### 3.6. Effects of CaHA on Genes Associated with Mitochondrial Stress Response and Energy Metabolism

Gene expression analysis of markers associated with mitochondrial biology, stress response, and cellular energy metabolism was performed to evaluate transcript-level responses to CaHA formulations in aging-like fibroblasts. Exposure to CaHA modulated the expression of genes involved in mitochondrial biogenesis, antioxidant defense, bioenergetic pathways, and mitochondrial quality-control mechanisms, including *PGC-1α*, *ATP5A1*, *SOD2*, and *PINK1*.

**Figure 6:**
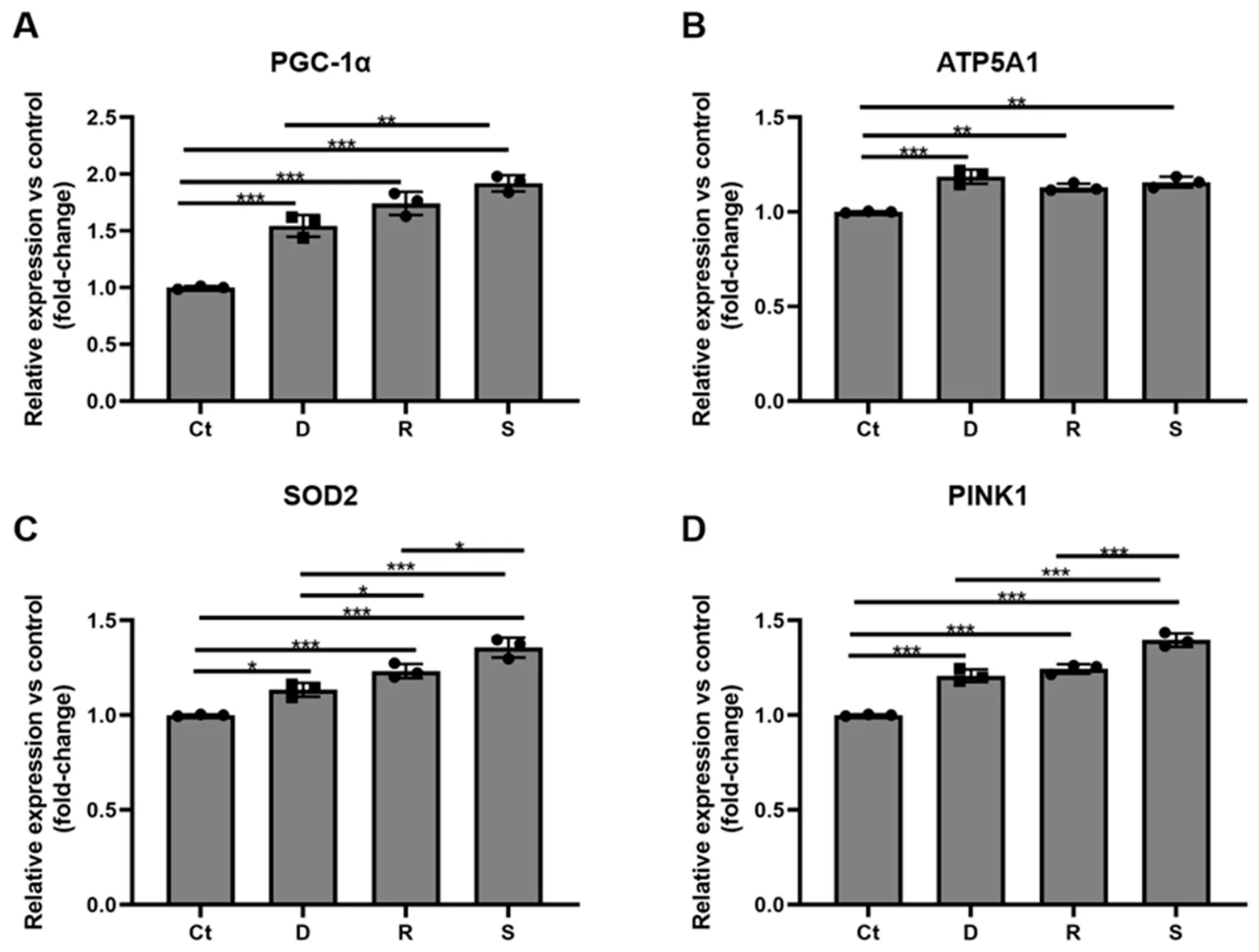
Relative gene expression of markers associated with mitochondrial stress response and energy metabolism across groups. (**A**) *PGC-1α*, (**B**) *ATP5A1*, (**C**) *SOD2*, and (**D**) *PINK1*. Data are shown as fold-change relative to the untreated control (normalized to 1.0). Horizontal bars indicate statistically significant differences between groups (* *p* < 0.05; ** *p* < 0.01; *** *p* < 0.001).

*PGC-1α* expression increased in all treatment groups, with sample S demonstrating the highest upregulation (S: +92.5 ± 7.2%; R: +72.6 ± 8.8%; D: +54.2 ± 9.1%). All treatments differed significantly from the control group, with a significant difference also observed between samples D and S (Figure 6A).

*ATP5A1* expression showed a moderate increase in all treated groups (D: +18.2 ± 5.2%; S: +15.8 ± 2.8%; R: +13.8 ± 2.2%), with significant differences relative to the control. However, no consistent pattern of differential expression among formulations was observed (Figure 6B).

*SOD2* expression increased in a treatment-dependent manner, with sample S exhibiting the highest induction (+35.2 ± 5.4%), followed by sample R (+23.4 ± 3.2%) and sample D (+12.8 ± 3.4%). Significant differences were observed both between the control and treated groups and among the different formulations (Figure 6C).

Similarly, *PINK1* expression was upregulated across all groups, with sample S showing the highest increase (+38.9 ± 2.9%), followed by sample R (+23.8 ± 2.3%) and sample D (+20.3 ± 2.2%). Expression in sample S was significantly higher than in both R and D (Figure 6D).

Collectively, these findings indicate that CaHA formulations differentially modulated the expression of genes associated with mitochondrial stress response, quality control, and energy metabolism in aging-like fibroblasts, with sample S showing the highest transcriptional response for *PGC-1α*, *SOD2*, and *PINK1*.

## 4. Discussion

CaHA-based fillers are recognized in aesthetic medicine for their dual-action mechanism, providing immediate volume replacement via a carrier gel and long-term biostimulatory effects mediated by fibroblast activation and neocollagenesis [15,17,28,29]. However, growing evidence suggests that their biological activity extends beyond these structural effects, encompassing broader modulation of cellular behavior [13,17,30,31]. Recent systematic-review evidence on HA/CaHA combined and hybrid approaches also supports the interpretation of CaHA-containing treatments through both immediate volumizing and longer-term biostimulatory mechanisms, although available clinical and mechanistic evidence remains heterogeneous [32].

In this context, the present study used an induced senescent human dermal fibroblast model to compare three commercial CaHA formulations across pathways associated with ECM remodeling, proliferative activity, inflammatory signaling, mitochondrial response, and longevity-associated gene regulation. Although this model does not capture the full spectrum of senescence markers, it provides a relevant functional platform to investigate fibroblast responses in an aging-like context. This cautious interpretation is consistent with recent work showing that different senescence-inducing stress patterns in human dermal fibroblasts may generate distinct transcriptional, secretory, and functional phenotypes [33].

Dermal aging skin is characterized by extracellular matrix degradation, impaired fibroblast–matrix interaction, and the accumulation of senescent fibroblasts with limited proliferative and biosynthetic capacity [3,6,7,34]. Because fibroblast function depends on mechanical anchorage to an organized extracellular matrix, deterioration of collagen architecture contributes to a self-reinforcing cycle of reduced tension sensing, lower collagen production, and defective tissue maintenance [3,5,35]. Within this context, CaHA microspheres may be interpreted as a local mechanostructural stimulus that provides surface contact for fibroblast interaction, potentially modulating cellular responses associated with matrix activation. The extent to which this contact reproduces the mechanical signaling provided by an organized ECM remains to be determined [17,19].

In the present study, all three tested CaHA formulations induced marked upregulation of collagen type I expression, with the strongest induction observed for samples S and R, while elastin upregulation was more selective and particularly pronounced for sample S. These findings indicate that CaHA exposure is associated with transcript-level changes across multiple gene categories beyond fibrillar collagen. However, these results should not be interpreted as direct evidence of protein-level ECM remodeling or functional cell–material effects. This interpretation is also aligned with the concepts of the literature, which describes CaHA-associated increases not only in collagen deposition but also in neoelastinogenesis, dermal thickening, and broader extracellular matrix remodeling [36,37]. Importantly, the differential magnitude of response across formulations suggests that commercially available CaHA products should not necessarily be assumed to be biologically equivalent at the cellular level, even when they belong to the same biomaterial class.

The proliferative findings reinforce this interpretation. *Ki67* is used as a marker of cellular proliferative activity and is classically described as being expressed throughout the active phases of the cell cycle and absent in quiescent cells, although its expression is now understood to be graded rather than strictly binary [38]. In our model, all formulations increased *Ki67* expression relative to the untreated EX-527-exposed control, with sample S showing the highest response. However, because *Ki67* was assessed only at the mRNA level, without *Ki67* immunostaining or functional proliferation assays, increased *Ki67* expression should be interpreted as a cell-cycle-associated transcriptional response rather than direct evidence of restored proliferative competence. It should also be acknowledged that the increase in *Ki67* expression observed in an EX-527-induced aging-like phenotype is biologically incongruent with the strict definition of senescence as an irreversible cell-cycle arrest. This may reflect either the partial nature of the EX-527-induced phenotype, heterogeneity within the cell population, or the absence of full quantitative and multimarker senescence validation, since SA-β-gal staining was included only as qualitative phenotypic support and additional canonical markers such as p16INK4A, p21CIP1, γH2AX, lamin B1, and broader SASP profiling were not assessed. This should temper any inference of proliferative rescue.

Inflammation represents another key dimension of dermal aging and tissue remodeling. Aged skin is increasingly understood as a site of chronic low-grade inflammatory activity, and senescent cells contribute to this state through the senescence-associated secretory phenotype, which includes cytokines, chemokines, growth factors, and matrix-remodeling mediators capable of perpetuating tissue dysfunction [6,7,8]. In parallel, biomaterial implantation in vivo initiates a sequence of events that include protein adsorption, provisional matrix formation, acute inflammation, monocyte/macrophage recruitment, and subsequent wound-healing responses [39,40]. Although the present model isolates fibroblast responses and does not fully reproduce the cascade, it remains useful for identifying fibroblast-intrinsic differences across formulations. Recent calcium phosphate microsphere data further indicate that particle size can influence macrophage polarization and cytokine secretion patterns, supporting the relevance of particle-related features in shaping biomaterial-associated inflammatory responses [41].

Within this context, the cytokine profile provides important insight into the qualitative nature of the cellular response induced by the tested formulations, as *IL-6* and *TNF-α* contribute to the pro-inflammatory environment associated with fibroblast dysfunction, whereas *IL-10* and TGF-β may play regulatory roles that, depending on the context, favor resolution and tissue repair [7,42,43]. The balance between pro-inflammatory and pro-regenerative immune signals is a key determinant of whether a biomaterial-induced response supports effective tissue remodeling or leads to chronic inflammation and impaired healing [8,43,44].

In the present study, the three CaHA formulations elicited markedly distinct cytokine profiles. All formulations modestly increased *IL-6* expression, but the most discriminative response was observed for *TNF-α*, which increased with sample D but decreased with samples R and S. At the same time, all formulations increased *IL-10* and TGF-β, with sample S showing the strongest *IL-10* response and sample R the highest TGF-β induction. Taken together, these transcript-level data suggest that samples R and S were associated with a more regulated cytokine gene-expression profile, whereas sample D exhibited a comparatively more pro-inflammatory transcriptional pattern. This distinction may be biologically relevant because persistent *TNF-α*-dominant signaling has been associated with impaired matrix synthesis and enhanced catabolic activity, whereas *IL-10*- and TGF-β-associated environments are more compatible with matrix deposition and repair-oriented remodeling [45,46]. At the same time, the increase in *IL-6* should not automatically be interpreted as detrimental, since transient *IL-6* signaling may also participate in early reparative or adaptive responses [39,40].

Although previous studies have explored the effects of CaHA on selected longevity-associated pathways [20], these responses remain only partially characterized, particularly when considered alongside mitochondrial function. In this context, the present study provides transcript-level information on mitochondrial-associated markers in aging-like dermal fibroblasts. These findings should be interpreted as preliminary and exploratory, as they are limited to transcript-level observations in a single in vitro model. Even so, samples R and S consistently induced substantially greater *SIRT1* upregulation (+37.4% and +35.4%, respectively) than sample D (+5.9%), while sample S showed the highest induction of *FOXO3* (+41.4%), *SIRT3* (+20.0%), *PGC-1α* (+92.5%), *SOD2* (+35.2%), and *PINK1* (+38.9%). The higher expression of *SOD2* and *PINK1*, particularly in sample S, may reflect activation of antioxidant and mitochondrial quality-control responses, but may also represent a transcriptional response to mitochondrial stress. Therefore, in the absence of functional mitochondrial assays, these findings should not be interpreted as direct evidence of improved mitochondrial function. Whether these transcriptional changes reflect a direct consequence of cell–material interaction, an indirect effect mediated by the altered inflammatory milieu, or a downstream response to improved cellular energetics remains to be determined.

Across all evaluated gene categories, sample S consistently demonstrated the most pronounced transcriptional responses, particularly in markers associated with cellular proliferation, elastin synthesis, longevity-related signaling, and mitochondrial function, while sample R showed higher *COL1A1* and *TGFB1* transcript-level responses; sample D, in contrast, was associated with a comparatively more pro-inflammatory profile and more modest induction of anabolic and cytoprotective genes.

The mechanistic basis for these formulation-dependent differences was not directly investigated in the present study. Direct contact between CaHA microspheres and fibroblasts has been reported to stimulate neocollagenesis [19]. Commercial CaHA-based biostimulators used in facial filling procedures have been shown to differ in particle size and morphology, despite presenting CaHA as a common inorganic component [47]. In a side-by-side in vitro comparison of two CaHA-based dermal biostimulators, differences in fibroblast metabolic activity and collagen/elastin gene expression were observed together with qualitative differences in microsphere surface microtopography [48]. Hydroxyapatite particle size and morphology have been shown to modulate inflammatory responses after implantation, including NLRP3 inflammasome activation, IL-1β secretion, cytokine production, and immune-cell recruitment [21]. Specific surface area may also influence cellular responses to hydroxyapatite particles, as rod-shaped hydroxyapatite nanoparticles with higher specific surface area showed greater cell–particle interaction and increased ROS generation in vitro [49]. Therefore, although particle size, morphology, surface microtopography, and surface area are plausible contributors to formulation-dependent biological responses, the present study cannot attribute the observed transcriptional differences to any specific physicochemical feature because samples D, R, and S were not directly characterized by SEM, particle-size distribution, BET surface-area analysis, rheology, or other material-testing methods. Dedicated comparative physicochemical studies will be required to determine whether the observed biological divergence is related to microsphere morphology, surface properties, carrier composition, or other formulation-specific characteristics [16,30]. The present findings should be interpreted in light of several important limitations. First, the results are based exclusively on transcript-level data obtained at a single non-cytotoxic concentration and a single 72-h time point in a two-dimensional in vitro model, without dose–response analysis, temporal characterization, corresponding protein-level validation by Western blot, ELISA, or immunostaining, or functional assays. Accordingly, the observed gene expression changes should be interpreted as indicative of pathway modulation rather than definitive evidence of functional biological effects. Second, the use of EX-527 to induce an aging-like fibroblast phenotype represents a pharmacological *SIRT1*-inhibition model and does not constitute a fully validated senescence system. Although representative SA-β-gal staining was included as qualitative phenotypic support, quantitative SA-β-galactosidase activity and additional canonical markers such as p16/p21 expression, DNA damage markers, or broader SASP profiling were not assessed. Therefore, the model should be interpreted as a *SIRT1*-inhibition-associated, aging-like cellular state rather than a definitive senescence model. Third, although differential biological responses were observed among the CaHA formulations, no direct physicochemical characterization (e.g., particle size distribution, surface morphology, or rheological properties) was performed, and the mechanistic basis for formulation-dependent effects remains speculative. Fourth, the observed increase in *Ki67* expression in a senescence-associated context should be interpreted with caution, as it may reflect partial restoration of proliferative signaling or cellular heterogeneity rather than reversal of senescence. Finally, potential conflicts of interest should be considered when interpreting the results.

Despite these limitations, the data provide comparative evidence that commercially available CaHA formulations may differ in their capacity to modulate multiple biological pathways in senescence-associated dermal fibroblasts, supporting the concept that CaHA biostimulation extends beyond neocollagenesis to encompass inflammatory regulation and, as a preliminary observation, pathways associated with cellular longevity and mitochondrial homeostasis.

Further studies incorporating protein-level validation, mitochondrial functional assays (e.g., respirometry and reactive oxygen species quantification), and three-dimensional or in vivo models will be necessary to confirm the biological significance of these findings and to clarify the mechanistic basis underlying formulation-dependent differences.

## 5. Conclusions

The present study shows that CaHA-based formulations differ in their ability to modulate gene expression of markers associated with multiple biological pathways in an aging-like dermal fibroblast model. Beyond type I collagen induction, the evaluated materials were associated with differential expression of genes related to proliferative activity, inflammatory signaling, longevity-associated pathways, and mitochondrial homeostasis. In this comparative in vitro analysis, sample S exhibited the broadest transcriptional response across the evaluated markers, sample R showed comparable activity for collagen type I and TGF-β expression, and sample D produced more modest responses with a relatively more pro-inflammatory cytokine gene-expression profile. Whether these in vitro transcriptional differences correspond to protein-level changes, functional biological effects, or clinically meaningful tissue-level outcomes cannot be inferred from this dataset and requires confirmation in protein-level, functional, and ideally in vivo studies.

## Figures and Tables

**Table 1:** Classification of gene expression markers according to biological function. Genes were grouped into structural matrix, cellular renewal, inflammatory response, longevity pathways, and mitochondrial function/energy metabolism categories for RT-qPCR analysis.

Biological Function Category	Genes Evaluated
Structural Matrix	*COL1A1* (collagen type I), *ELN* (elastin)
Cellular Renewal	*Ki67*
Longevity Pathways	*FOXO3*, *SIRT1*, *SIRT3*
Pro-inflammatory Response	*IL-6*, *TNF-α*
Anti-inflammatory and regulatory Response	*IL10*, *TGFB1* (TGF-β1)
Mitochondrial Health and Energy	*PGC-1α*, *ATP5A1*, *SOD2*, *PINK1*

## Data Availability

The data presented in this study are available on request from the corresponding author due to the product-specific nature of the comparative experimental analyses. The raw and processed data supporting the central findings of this study include RT-qPCR Ct/ΔCt/ΔΔCt values, fold-change calculations, group-level summaries, and statistical outputs used to generate the figures and analyses. No personally identifiable information is included in the dataset. No custom code or scripts were generated for the analyses.

## Supplementary Materials

The following supporting information can be downloaded at: https://www.mdpi.com/article/10.3390/life16091400/s1, Table S1: Primer sequences used for quantitative real-time PCR (RT-qPCR) analysis.

## Abbreviations

The following abbreviations are used in this manuscript:

*ACTB*	Beta-actin
*ATP5A1*	ATP synthase F1 subunit alpha
CaHA	Calcium hydroxylapatite
cDNA	Complementary DNA
*COL1A1*	Collagen type I alpha 1 chain
DMEM	Dulbecco’s Modified Eagle’s Medium
ECM	Extracellular matrix
*ELN*	Elastin
EX-527	SIRT1 inhibitor; Selisistat
FBS	Fetal bovine serum
*FOXO3*	Forkhead box O3
*IL-6*	Interleukin-6
*IL-10*	Interleukin-10
IRB	Institutional Review Board
*Ki67*	Marker of proliferation Ki-67
NAD^+^	Nicotinamide adenine dinucleotide
*PGC-1α*	Peroxisome proliferator-activated receptor gamma coactivator 1-alpha
*PINK1*	PTEN-induced kinase 1
RNA	Ribonucleic acid
ROS	Reactive oxygen species
RT-qPCR	Quantitative reverse transcription polymerase chain reaction
SA-β-gal	Senescence-associated beta-galactosidase
*SIRT1*	Sirtuin 1
*SIRT3*	Sirtuin 3
*SOD2*	Superoxide dismutase 2
*TGFB1*	Transforming growth factor beta 1 gene
TGF-β	Transforming growth factor beta
*TNF-α*	Tumor necrosis factor alpha

## References

[1] Fisher G.J., Kang S., Varani J., Bata-Csorgo Z., Wan Y., Datta S., Voorhees J.J. (2002). Mechanisms of Photoaging and Chronological Skin Aging. Arch. Dermatol..

[2] Quan T., Fisher G.J. (2015). Role of Age-Associated Alterations of the Dermal Extracellular Matrix Microenvironment in Human Skin Aging: A Mini-Review. Gerontology.

[3] Varani J., Dame M.K., Rittie L., Fligiel S.E.G., Kang S., Fisher G.J., Voorhees J.J. (2006). Decreased collagen production in chronologically aged skin: Roles of age-dependent alteration in fibroblast function and defective mechanical stimulation. Am. J. Pathol..

[4] Fisher G.J., Varani J., Voorhees J.J. (2008). Looking Older: Fibroblast collapse and therapeutic implications. Arch. Dermatol..

[5] Varani J., Schuger L., Dame M.K., Leonard C., Fligiel S.E.G., Kang S., Fisher G.J., Voorhees J.J. (2004). Reduced Fibroblast Interaction with Intact Collagen as a Mechanism for Depressed Collagen Synthesis in Photodamaged Skin. J. Investig. Dermatol..

[6] Low E., Alimohammadiha G., Smith L.A., Costello L.F., Przyborski S.A., von Zglinicki T., Miwa S. (2021). How good is the evidence that cellular senescence causes skin ageing?. Ageing Res. Rev..

[7] Zhang J., Yu H., Man M.Q., Hu L. (2024). Aging in the dermis: Fibroblast senescence and its significance. Aging Cell.

[8] Agrawal R., Hu A., Bollag W.B. (2023). The Skin and Inflamm-Aging. Biology.

[9] Nan L., Guo P., Hui W., Xia F., Yi C. (2025). Recent advances in dermal fibroblast senescence and skin aging: Unraveling mechanisms and pioneering therapeutic strategies. Front. Pharmacol..

[10] Thau H., Gerjol B.P., Hahn K., von Gudenberg R.W., Knoedler L., Stallcup K., Emmert M.Y., Buhl T., Wyles S.P., Tchkonia T. (2025). Senescence as a molecular target in skin aging and disease. Ageing Res. Rev..

[11] Herranz D., Muñoz-Martin M., Cañamero M., Mulero F., Martinez-Pastor B., Fernandez-Capetillo O., Serrano M. (2010). Sirt1 improves healthy ageing and protects from metabolic syndrome-associated cancer. Nat. Commun..

[12] Rogina B., Tissenbaum H.A. (2024). SIRT1, resveratrol and aging. Front. Genet..

[13] Aguilera S.B., McCarthy A., Khalifian S., Lorenc Z.P., Goldie K., Chernoff W.G. (2023). The Role of Calcium Hydroxylapatite (Radiesse) as a Regenerative Aesthetic Treatment: A Narrative Review. Aesthetic Surg. J..

[14] De Almeida A.T., Figueredo V., Da Cunha A.L.G., Casabona G., Costa De Faria J.R., Alves E.V., Sato M., Branco A., Guarnieri C., Palermo E. (2019). Consensus Recommendations for the Use of Hyperdiluted Calcium Hydroxyapatite (Radiesse) as a Face and Body Biostimulatory Agent. Plast. Reconstr. Surg.-Glob. Open.

[15] Goldie K., Peeters W., Alghoul M., Butterwick K., Casabona G., Chao Y.Y.Y., Costa J., Eviatar J., Fabi S.G., Lupo M. (2018). Global Consensus Guidelines for the Injection of Diluted and Hyperdiluted Calcium Hydroxylapatite for Skin Tightening. Dermatol. Surg..

[16] Karatas E., Koc K., Yilmaz M., Aydin H.M. (2024). Characterization and Comparative Investigation of Hydroxyapatite/Carboxymethyl Cellulose (CaHA/CMC) Matrix for Soft Tissue Augmentation in a Rat Model. ACS Omega.

[17] Van Loghem J. (2024). Calcium Hydroxylapatite in Regenerative Aesthetics: Mechanistic Insights and Mode of Action. Aesthetic Surg. J..

[18] Liu S., Song L., Huang S., Liu Z., Xu Y., Wang Z., Qiu H., Wang J., Chen Z., Xiao Y. (2025). Hydroxyapatite microspheres encapsulated within hybrid hydrogel promote skin regeneration through the activation of Calcium Signaling and Motor Protein pathway. Bioact. Mater..

[19] Nowag B., Casabona G., Kippenberger S., Zöller N., Hengl T. (2023). Calcium hydroxylapatite microspheres activate fibroblasts through direct contact to stimulate neocollagenesis. J. Cosmet. Dermatol..

[20] Dal Col V., Marchi C., Ribas F., Franzen Matte B., Medeiros H., Domenici de Oliveira B., Viana R. (2025). In Vitro Comparative Study of Calcium Hydroxyapatite (Stiim): Conventional Saline Dilution Versus Poly-Micronutrient Dilution. Cureus.

[21] Lebre F., Sridharan R., Sawkins M.J., Kelly D.J., O’Brien F.J., Lavelle E.C. (2017). The shape and size of hydroxyapatite particles dictate inflammatory responses following implantation. Sci. Rep..

[22] Denizot F., Lang R. (1986). Rapid colorimetric assay for cell growth and survival Modifications to the tetrazolium dye procedure giving improved sensitivity and reliability. J. Immunol. Methods.

[23] Mosmann T. (1983). Rapid colorimetric assay for cellular growth and survival: Application to proliferation and cytotoxicity assays. J. Immunol. Methods.

[24] Gertz M., Fischer F., Nguyen G.T.T., Lakshminarasimhan M., Schutkowski M., Weyand M., Steegborn C. (2013). Ex-527 inhibits Sirtuins by exploiting their unique NAD^+^-dependent deacetylation mechanism. Proc. Natl. Acad. Sci. USA.

[25] Napper A.D., Hixon J., McDonagh T., Keavey K., Pons J.F., Barker J., Yau W.T., Amouzegh P., Flegg A., Hamelin E. (2005). Discovery of indoles as potent and selective inhibitors of the deacetylase SIRT1. J. Med. Chem..

[26] Solomon J.M., Pasupuleti R., Xu L., McDonagh T., Curtis R., DiStefano P.S., Huber L.J. (2006). Inhibition of SIRT1 Catalytic Activity Increases p53 Acetylation but Does Not Alter Cell Survival following DNA Damage. Mol. Cell. Biol..

[27] Dimri G.P., Lee X., Basile G., Acosta M., Scott G., Roskelley C., Medrano E.E., Linskens M., Rubelji I., Pereira-Smith O. (1995). A biomarker that identifies senescent human cells in culture and in aging skin in vivo. Proc. Natl. Acad. Sci. USA.

[28] Hong J.Y., Park K.Y. (2025). Dual Benefits of Calcium Hydroxyapatite Filler: A Prospective Study on Midface Volume Restoration and Skin Quality Enhancement. J. Cosmet. Dermatol..

[29] Van Loghem J., Yutskovskaya Y.A., Philip Werschler W. (2015). Calcium hydroxylapatite: Over a decade of clinical experience. J. Clin. Aesthetic Dermatol..

[30] Amiri M., Meçani R., Niehot C.D., Phillips T., Kolb J., Daughtry H., Muka T. (2023). Skin regeneration-related mechanisms of Calcium Hydroxylapatite (CaHA): A systematic review. Front. Med..

[31] Carruthers J., Burgess C., Day D., Fabi S.G., Goldie K., Kerscher M., Nikolis A., Pavicic T., Rho N.-K., Rzany B. (2016). Consensus Recommendations for Combined Aesthetic Interventions in the Face Using Botulinum Toxin, Fillers, and Energy-Based Devices. Dermatol. Surg..

[32] Meçani R., Amiri M., Kadouch J., Sajic D., Lin F., Cheung J., Barrera D., Haroon O., Sil-Zavaleta S., Chao Y. (2025). Combined and Hybrid Treatments of Hyaluronic Acid (HA) and Calcium Hydroxylapatite (CaHA): A Systematic Review of Mechanisms of Action, Aesthetic Effectiveness, Satisfaction, and Safety Profile. Aesthetic Plast. Surg..

[33] McCarty T.Y., Kearney C.J. (2025). Human dermal fibroblast senescence in response to single and recurring oxidative stress. Front. Aging.

[34] Friedman O. (2005). Changes Associated with the Aging Face. Facial Plast. Surg. Clin. N. Am..

[35] Carlson M.A., Smith L.M., Cordes C.M., Chao J., Eudy J.D. (2013). Attachment-regulated signaling networks in the fibroblast-populated 3D collagen matrix. Sci. Rep..

[36] Goldie K. (2023). The evolving field of regenerative aesthetics. J. Cosmet. Dermatol..

[37] Zarbafian M., Fabi S.G., Dayan S., Goldie K. (2022). The Emerging Field of Regenerative Aesthetics—Where We Are Now. Dermatol. Surg..

[38] Uxa S., Castillo-Binder P., Kohler R., Stangner K., Müller G.A., Engeland K. (2021). Ki-67 gene expression. Cell Death Differ..

[39] Anderson J.M., Rodriguez A., Chang D.T. (2008). Foreign body reaction to biomaterials. Semin. Immunol..

[40] Sheikh Z., Brooks P., Barzilay O., Fine N., Glogauer M. (2015). Macrophages, Foreign Body Giant Cells and Their Response to Implantable Biomaterials. Materials.

[41] Wan Q., Tian L., Wang M., Chen F., Li X., Xiao Y., Chen X., Zhang X. (2024). Immunomodulatory effects of calcium phosphate microspheres: Influences of particle size on macrophage polarization and secretion patterns. J. Mater. Chem. B.

[42] Kumari R., Jat P. (2021). Mechanisms of Cellular Senescence: Cell Cycle Arrest and Senescence Associated Secretory Phenotype. Front. Cell Dev. Biol..

[43] Nowag B., Schäfer D., Hengl T., Corduff N., Goldie K. (2024). Biostimulating fillers and induction of inflammatory pathways: A preclinical investigation of macrophage response to calcium hydroxylapatite and poly-L lactic acid. J. Cosmet. Dermatol..

[44] Biglari N., Razzaghi M., Afkham Y., Azimi G., Gross J.D., Samadi A. (2025). Advanced biomaterials in immune modulation: The future of regenerative therapies. Int. J. Pharm..

[45] Greenwel P., Tanaka S., Penkov D., Zhang W., Olive M., Moll J., Vinson C., Di Liberto M., Ramirez F. (2000). Tumor necrosis factor alpha inhibits type I collagen synthesis through repressive CCAAT/enhancer-binding proteins. Mol. Cell. Biol..

[46] Han Y.P., Tuan T.L., Wu H., Hughes M., Garner W.L. (2001). TNF-alpha stimulates activation of pro-MMP2 in human skin through NF-(kappa)B mediated induction of MT1-MMP. J. Cell Sci..

[47] de Moraes Nobre M., Fraga M.A.A., Dal Coll V., Brito C.A., de Menezes L.R., Thebit M.M., Correr A.B. (2025). Characterization of Particles From Collagen Bio-Stimulating Materials Used in Facial Filling Procedures. Microsc. Res. Tech..

[48] Col V.D., Matte B.F. (2026). Calcium Hydroxyapatite Biostimulators: A Comparative Study of Biological Response and Particle Morphology. Biomedicines.

[49] Zhao X., Heng B.C., Xiong S., Guo J., Tan T.T.Y., Boey F.Y.C., Ng K.W., Loo J.S.C. (2011). In vitro assessment of cellular responses to rod-shaped hydroxyapatite nanoparticles of varying lengths and surface areas. Nanotoxicology.

