# Supplementary material for: Differential Modulation of Extracellular Matrix- and Longevity-Related Gene Expression by Calcium Hydroxylapatite Formulations
Source: Life (Basel). 2026 Aug 25;16(9):1400. doi: 10.3390/life16091400 (PMC13608777; doi:10.3390/life16091400)
Supplement: Supplementary file 1 [file life-16-01400-s001.zip › life-4410045-supplementary.pdf]

Supplementary Table S1

Table S1. Primer sequences used for quantitative real-time PCR (RT-qPCR) analysis. Forward and reverse primer sequences used to assess the expression of genes associated with extracellular matrix remodeling, cellular proliferation, inflammatory and regulatory signaling, mitochondrial function, bioenergetics, and longevity-associated pathways. All primer sequences are presented in the 5′–3′ direction.

**Target gene**	**Forward primer (5′–3′)**	**Reverse primer (5′–3′)**
ATP5A1	GTATTGCCCGCGTACATGG	AGGACATACCCTTTAAGCCTGA
COL1A1	AGGGCCAAGACATC	AGATCACGTCATCGCACAACA
Elastin	GTATATACCCAGGTGGAGTG	CGAACTTTGCTGCTGCTTTAG
FoxO3	AGAACTCCATCCGGCACA	TATCACTCAGCCGTGGCA
IL-10	ACGGCCTTGCTCTTGTTTTC	AAGGCGCATGTGAACTCCC
IL-6	CTGGCTTGTTCCTCACTAC	ATGGATGCTTCCAATCTG
Ki67	GGGCCAATCCTGTCGCTTAAT	GTTATGCGCTTGCGAACCT
PGC-1α	AGCCGTGACCACTGACAACG	GTCGCATGGTTCTGAGTGCTAG
PINK1	TGGAGGAGGACTACGACAGC	CAGGATGAGGTTGTCGTTGG
SIRT-1	GCCTCACATGCAAGCTCTAGTGAC	TTCGAGGATCTGTGCCAATCATAA
SIRT-3	CTTGTGCAGCGGGAAACT	TCCATAGTTACCATTTATTGTGTGG
SOD2	CAGACCTGCCTTACGACTATGG	CTCGGTGGCGTTGAGATTG
TGFB1	AGTGTGTTATCCCTGCTGTCAC	GCCAGAGTGGTTATCTTTTGATG
TNF-α	CCAGAGGGAAGAGCAGTCC	GGCTACAACGTGGGCTACC
